# Supplementary material for: DNA Vaccine Co-Expressing Hemagglutinin and IFN-γ Provides Partial Protection to Ferrets against Lethal Challenge with Canine Distemper Virus
Source: Viruses. 2023 Sep 4;15(9):1873. doi: 10.3390/v15091873 (PMC10537869; doi:10.3390/v15091873)
Supplement: Supplementary file 1 [file viruses-15-01873-s001.zip › S1 Codon optimization sequence for SD(14)7-H and comparison with parental sequence.pdf]

Optimization report

Gene name: SD(14)7-H
Gene length: 1824bp
Optimization region: 1 - 1824
Optimized system: Neovison vison

1. Optimized sequence

ATGCTGTCTTACCAGGACAAGGTGGGCGCTTTCTACAAGGACAACGCTAGAGCCAACAGTAGTAAGCTGTCTCTGGTGACCGAAGAGCAG
GGCGGACGCCGGCCCCCTTACCTGCTGCTCGTGCTCCTGTCTCTGCTGATTGGCATACTGACACTGCTCGCCATTACCGGGGTGCGGTTC
CACCAGGTCAGCACATCTAACATGGAGTTCAGTAGACTGCTGAAAGAGGACATGGAGAAGTCTGAGGCCGTGCACCACCAGGTTATTGAC
GTCCTGACCCCACTCTTCAAGATTATTGGGGACGAGATTGGCCTCAGGCTGCCCCAGAAGCTGAACGAGATCAAGCAGTTCATTCTCCAA
AAGACAAACTTCTTCAACCCTAACCGCGAGTTCGACTTCCGCGACCTGCACTGGTGCATTAACCCCCCTAGTAAGATCAAGGTCAACTTC
ACAAACTACTGCGACACCGTCGGGGTCAAGAAGTCTATCGCAAGTGCCGTGAACCCTATTATTCTGTCCGCTCTCAGTGGCGCTAGAGGC
GACATTTTCCCTCCATACAGATGCAGCGGGGCTACAACAAGTGTGGGTCGCGTGTTCAGCCTCAGCGTGAGCCTGTCTATGAGCCTGATT
TCCCGCACAAGTGAGATTATCAACATGCTGACAGCTATTAGTGACGGGGTGTACGGAAAGACATACCTCCTGGTGCCCGACTACATTGAG
GGCGAGTTCGACTCTCAGAAGATCCGCGTGTTCGAGATCGGATTCATTAAGCGGTGGCTGAACAACATCCCACTCCTTCAAACAACAAAC
TACATGGTCCTCCCCGAGACCAGCAAGGCTAAGGTGTGCACCATCGCCGTCGGCGAACTCACACTCGCTAGTCTGTGCGTCGATGAGAGT
ACAGTCCTCCTGTACCACGACTCTAACGGAAGTCAGAACGGCATCCTCGTCGTCACACTGGGAATTTTCGGCGCCACCCCTATGGACCAG
GTGGAAGAAGTCATCCCTATCGCCCACCCATCCGTCGAGCGAATCCACATTACAAACCACCGCGGGTTCATTAAGGACTCTGTGGTCACC
TGGATGGTCCCCGTCCTGGTCAGTGAGAAGCAAGAAGAGCAGAAGAACTGCCTCGAAAGCGCTTGCCAGCGCAAGAGTTACCCAATGTGC
AACCAGACATCTTGGGAGCCTTTCGGCGGCGGGCAGCTGCCTAGTTACGGCAGGCTCACACTGCCTCTCGACCCATCTGTGGACCTCCAG
CTCAACATTAGCTTCACATACGGGCCCGTGATTCTGAACGGGGACGGCATGGACTACTACGAGAGTCCACTCCTGGAGTCCGGATGGCTC
ACAATCCCTCCAAAGAACGGAACAGTGCTGGGACTGATTAACAAGGCTAGTAGGGGCGACCAGTTCACAGTGACACCTCACGTCCTGACC
TTCGCCCCTAGGGAGTCTAGCGGAAACTGCTACCTGCCAATCCAGACATCTCAGATCATGGACAAGGACGTCCTCACCGAGAGTAACCTC
GTCGTGCTCCCAACACAGAACTTCCGCTATGTCATCGCTACATACGACATTAGTAGGGGAGACCACGCTATCGTGTACTATGTCTACGAC
CCTAACCGCACAATTTCTTACACCCACCCTTTCCGGCTCACCACAAAGGGCAGGCCCGACTTCCTGCGCATTGAGTGCTTCGTGTGGGAC
GACGACCTGTGGTGCCACCAGTTCTACCGGTTCGAGGCCAACATCACAAACTCTACCACAAGTGTCGAGAACCTCGTGCGCATCCGGTTC
TCTTGCAACCGGTCTAAGCCATAG

2. DNA Alignment

```
Optimized       1    ATGCTGTCTTACCAGGACAAGGTGGGCGCTTTCTACAAGGACAACGCTAGAGCCAACAGT
Original        1    ATGCTCTCCTACCAAGACAAGGTGGGTGCCTTCTATAAGGATAATGCAAGAGCTAATTCA

Optimized      61    AGTAAGCTGTCTCTGGTGACCGAAGAGCAGGGCGGACGCCGGCCCCCTTACCTGCTGCTC
Original       61    TCCAAGCTGTCCTTAGTGACAGAAGAGCAAGGGGGAAGGAGACCACCCTATTTGCTGCTT

Optimized     121    GTGCTCCTGTCTCTGCTGATTGGCATACTGACACTGCTCGCCATTACCGGGGTGCGGTTC
Original      121    GTCCTTCTCAGCCTACTGATTGGAATCCTGACCTTGCTTGCCATCACTGGAGTTCGATTT

Optimized     181    CACCAGGTCAGCACATCTAACATGGAGTTCAGTAGACTGCTGAAAGAGGACATGGAGAAG
Original      181    CACCAAGTATCAACTAGCAATATGGAATTTAGCAGATTGCTGAAAGAGGATATGGAGAAA

Optimized     241    TCTGAGGCCGTGCACCACCAGGTTATTGACGTCCTGACCCCACTCTTCAAGATTATTGGG
Original      241    TCAGAGGCCGTACATCACCAAGTCATAGATGTCCTGACACCGCTCTTCAAAATTATTGGA

Optimized     301    GACGAGATTGGCCTCAGGCTGCCCCAGAAGCTGAACGAGATCAAGCAGTTCATTCTCCAA
Original      301    GATGAGATTGGGTTGCGGTTGCCACAAAAACTAAACGAGATCAAACAATTTATCCTTCAA

Optimized     361    AAGACAAACTTCTTCAACCCTAACCGCGAGTTCGACTTCCGCGACCTGCACTGGTGCATT
Original      361    AAGACAAACTTCTTCAATCCGAACAGGGAATTCGACTTCCGCGATCTCCACTGGTGCATT

Optimized     421    AACCCCCCTAGTAAGATCAAGGTCAACTTCACAAACTACTGCGACACCGTCGGGGTCAAG
```

```
Original       421   AACCCACCTAGCAAGATCAAGGTGAATTTTACCAATTACTGTGATACAGTTGGGGTCAAA

Optimized      481   AAGTCTATCGCAAGTGCCGTGAACCCTATTATTCTGTCCGCTCTCAGTGGCGCTAGAGGC
Original       481   AAATCTATTGCATCGGCAGTAAATCCCATCATTTTATCAGCACTCTCCGGAGCCAGAGGC

Optimized      541   GACATTTTCCCTCCATACAGATGCAGCGGGGCTACAACAAGTGTGGGTCGCGTGTTCAGC
Original       541   GACATATTCCCGCCGTACAGATGCAGTGGAGCTACTACTTCAGTAGGCAGAGTATTCTCC

Optimized      601   CTCAGCGTGAGCCTGTCTATGAGCCTGATTTCCCGCACAAGTGAGATTATCAACATGCTG
Original       601   CTATCCGTGTCATTATCCATGTCTTTAATATCAAGAACATCAGAGATAATCAATATGCTA

Optimized      661   ACAGCTATTAGTGACGGGGTGTACGGAAAGACATACCTCCTGGTGCCCGACTACATTGAG
Original       661   ACCGCTATCTCAGACGGCGTGTATGGTAAAACTTATTTGCTAGTGCCTGATTATATTGAA

Optimized      721   GGCGAGTTCGACTCTCAGAAGATCCGCGTGTTCGAGATCGGATTCATTAAGCGGTGGCTG
Original       721   GGGGAGTTCGACTCGCAAAAGATTCGAGTCTTTGAGATAGGGTTTATCAAACGGTGGCTG

Optimized      781   AACAACATCCCACTCCTTCAAACAACAAACTACATGGTCCTCCCCGAGACCAGCAAGGCT
Original       781   AATAACATACCTTTACTCCAGACAACCAACTATATGGTCCTCCCGGAAACTTCCAAAGCC

Optimized      841   AAGGTGTGCACCATCGCCGTCGGCGAACTCACACTCGCTAGTCTGTGCGTCGATGAGAGT
Original       841   AAGGTATGTACTATAGCAGTGGGCGAGCTGACACTAGCTTCCTTGTGTGTAGATGAGAGC

Optimized      901   ACAGTCCTCCTGTACCACGACTCTAACGGAAGTCAGAACGGCATCCTCGTCGTCACACTG
Original       901   ACCGTATTGTTATATCATGACAGCAATGGTTCACAAAATGGTATTCTAGTAGTGACATTG

Optimized      961   GGAATTTTCGGCGCCACCCCTATGGACCAGGTGGAAGAAGTCATCCCTATCGCCCACCCA
Original       961   GGAATATTTGGGGCAACACCTATGGATCAAGTTGAAGAGGTGATACCTATCGCTCACCCA

Optimized     1021   TCCGTCGAGCGAATCCACATTACAAACCACCGCGGGTTCATTAAGGACTCTGTGGTCACC
Original      1021   TCAGTGGAGAGAATACATATAACAAATCACCGTGGGTTCATAAAAGATTCAGTAGTAACC

Optimized     1081   TGGATGGTCCCCGTCCTGGTCAGTGAGAAGCAAGAAGAGCAGAAGAACTGCCTCGAAAGC
Original      1081   TGGATGGTGCCTGTATTGGTCTCTGAGAAACAAGAGGAGCAAAAAAACTGTCTGGAGTCT

Optimized     1141   GCTTGCCAGCGCAAGAGTTACCCAATGTGCAACCAGACATCTTGGGAGCCTTTCGGCGGC
Original      1141   GCTTGTCAAAGAAAATCTTACCCGATGTGTAACCAAACGTCATGGGAACCCTTTGGAGGA

Optimized     1201   GGGCAGCTGCCTAGTTACGGCAGGCTCACACTGCCTCTCGACCCATCTGTGGACCTCCAG
Original      1201   GGACAGTTGCCCTCTTATGGGCGGTTGACATTACCTCTAGATCCAAGCGTTGACCTTCAA

Optimized     1261   CTCAACATTAGCTTCACATACGGGCCCGTGATTCTGAACGGGGACGGCATGGACTACTAC
Original      1261   CTTAACATATCATTTACATATGGTCCGGTTATACTGAACGGAGACGGTATGGATTATTAT

Optimized     1321   GAGAGTCCACTCCTGGAGTCCGGATGGCTCACAATCCCTCCAAAGAACGGAACAGTGCTG
Original      1321   GAAAGCCCACTTTTGGAATCCGGATGGCTAACCATACCCCCTAAGAACGGAACAGTCCTT

Optimized     1381   GGACTGATTAACAAGGCTAGTAGGGGCGACCAGTTCACAGTGACACCTCACGTCCTGACC
Original      1381   GGATTGATAAACAAAGCAAGTAGAGGAGACCAGTTCACTGTGACCCCCCATGTGTTGACA
```

```
Optimized    1441    TTCGCCCCTAGGGAGTCTAGCGGAAACTGCTACCTGCCAATCCAGACATCTCAGATCATG
Original     1441    TTTGCGCCCAGGGAATCAAGTGGAAATTGTTATTTGCCTATTCAAACATCCCAGATTATG

Optimized    1501    GACAAGGACGTCCTCACCGAGAGTAACCTCGTCGTGCTCCCAACACAGAACTTCCGCTAT
Original     1501    GATAAAGATGTCCTTACTGAGTCCAATTTAGTGGTGTTACCTACACAGAATTTTAGATAT

Optimized    1561    GTCATCGCTACATACGACATTAGTAGGGGAGACCACGCTATCGTGTACTATGTCTACGAC
Original     1561    GTCATAGCAACATATGATATATCCCGGGGCGATCATGCAATTGTTTATTATGTTTATGAC

Optimized    1621    CCTAACCGCACAATTTCTTACACCCACCCTTTCCGGCTCACCACAAAGGGCAGGCCCGAC
Original     1621    CCTAACCGGACGATTTCTTATACACACCCATTTAGGCTAACTACCAAGGGTAGACCTGAT

Optimized    1681    TTCCTGCGCATTGAGTGCTTCGTGTGGGACGACGACCTGTGGTGCCACCAGTTCTACCGG
Original     1681    TTCCTAAGGATTGAATGTTTTGTGTGGGATGACGATCTGTGGTGTCATCAATTTTACCGA

Optimized    1741    TTCGAGGCCAACATCACAAACTCTACCACAAGTGTCGAGAACCTCGTGCGCATCCGGTTC
Original     1741    TTCGAGGCTAACATCACTAACTCTACAACCAGTGTTGAGAATTTAGTCCGTATAAGATTC

Optimized    1801    TCTTGCAACCGGTCTAAGCCATAG
Original     1801    TCATGTAACCGTTCAAAACCTTGA
```

3. Supplementary - Codon frequency table

Species: Neovison vison

TTT	0.405	TTC	0.595	TTA	0.054	TTG	0.123
TCT	0.179	TCC	0.246	TCA	0.129	TCG	0.050
TAT	0.392	TAC	0.608	TAA	0.263	TAG	0.368
TGT	0.444	TGC	0.556	TGA	0.368	TGG	1.000
CTT	0.127	CTC	0.238	CTA	0.056	CTG	0.402
CCT	0.292	CCC	0.373	CCA	0.248	CCG	0.087
CAT	0.395	CAC	0.605	CAA	0.237	CAG	0.763
CGT	0.093	CGC	0.168	CGA	0.101	CGG	0.229
ATT	0.326	ATC	0.553	ATA	0.121	ATG	1.000
ACT	0.184	ACC	0.414	ACA	0.264	ACG	0.137
AAT	0.430	AAC	0.570	AAA	0.378	AAG	0.622
AGT	0.151	AGC	0.244	AGA	0.217	AGG	0.192
GTT	0.145	GTC	0.253	GTA	0.101	GTG	0.501
GCT	0.267	GCC	0.447	GCA	0.181	GCG	0.105
GAT	0.385	GAC	0.615	GAA	0.408	GAG	0.592
GGT	0.141	GGC	0.376	GGA	0.252	GGG	0.231

4. Supplementary - Protein Alignment

```
Optimized      1    MLSYQDKVGAFYKDNARANSSKLSLVTEEQGGRRPPYLLLVLLSLLIGILTLLAITGVRF
Original       1    MLSYQDKVGAFYKDNARANSSKLSLVTEEQGGRRPPYLLLVLLSLLIGILTLLAITGVRF

Optimized     61    HQVSTSNMEFSRLLKEDMEKSEAVHHQVIDVLTPLFKIIGDEIGLRLPQKLNEIKQFILQ
Original      61    HQVSTSNMEFSRLLKEDMEKSEAVHHQVIDVLTPLFKIIGDEIGLRLPQKLNEIKQFILQ

Optimized    121    KTNFFNPNREFDFRDLHWCINPPSKIKVNFTNYCDTVGVKKSIASAVNPIILSALSGARG
Original     121    KTNFFNPNREFDFRDLHWCINPPSKIKVNFTNYCDTVGVKKSIASAVNPIILSALSGARG

Optimized    181    DIFPPYRCSGATTSVGRVFSLSVSLSMSLISRTSEIINMLTAISDGVYGKTYLLVPDYIE
Original     181    DIFPPYRCSGATTSVGRVFSLSVSLSMSLISRTSEIINMLTAISDGVYGKTYLLVPDYIE

Optimized    241    GEFDSQKIRVFEIGFIKRWLNNIPLLQTTNYMVLPETSKAKVCTIAVGELTLASLCVDES
Original     241    GEFDSQKIRVFEIGFIKRWLNNIPLLQTTNYMVLPETSKAKVCTIAVGELTLASLCVDES

Optimized    301    TVLLYHDSNGSQNGILVVTLGIFGATPMDQVEEVIPIAHPSVERIHITNHRGFIKDSVVT
Original     301    TVLLYHDSNGSQNGILVVTLGIFGATPMDQVEEVIPIAHPSVERIHITNHRGFIKDSVVT

Optimized    361    WMVPVLVSEKQEEQKNCLESACQRKSYPMCNQTSWEPFGGGQLPSYGRLTLPLDPSVDLQ
Original     361    WMVPVLVSEKQEEQKNCLESACQRKSYPMCNQTSWEPFGGGQLPSYGRLTLPLDPSVDLQ

Optimized    421    LNISFTYGPVILNGDGMDYYESPLLESGWLTIPPKNGTVLGLINKASRGDQFTVTPHVLT
Original     421    LNISFTYGPVILNGDGMDYYESPLLESGWLTIPPKNGTVLGLINKASRGDQFTVTPHVLT

Optimized    481    FAPRESSGNCYLPIQTSQIMDKDVLTESNLVVLPTQNFRYVIATYDISRGDHAIVYYVYD
Original     481    FAPRESSGNCYLPIQTSQIMDKDVLTESNLVVLPTQNFRYVIATYDISRGDHAIVYYVYD

Optimized    541    PNRTISYTHPFRLTTKGRPDFLRIECFVWDDDLWCHQFYRFEANITNSTTSVENLVRIRF
Original     541    PNRTISYTHPFRLTTKGRPDFLRIECFVWDDDLWCHQFYRFEANITNSTTSVENLVRIRF

Optimized    601    SCNRSKP!
Original     601    SCNRSKP!
```
