# Supplementary material for: DNA Vaccine Co-Expressing Hemagglutinin and IFN-γ Provides Partial Protection to Ferrets against Lethal Challenge with Canine Distemper Virus
Source: Viruses. 2023 Sep 4;15(9):1873. doi: 10.3390/v15091873 (PMC10537869; doi:10.3390/v15091873)
Supplement: Supplementary file 1 [file viruses-15-01873-s001.zip › S2 Codon optimization sequence for SD(14)7-N and comparison with parental sequence.pdf]

Optimization report

Gene name: SD(14)7-N
Gene length: 1572bp
Optimization region: 1 - 1572
Optimized system: Neovison vison

1. Optimized sequence

```
ATGGCCAGTCTCCTGAAGAGCCTCACACTGTTCAAGCGCACACGCGACCAGCCACCACTGGCTTCCGGAAGTGGCGGGGCTATTAGGGGA
ATCAAGCACGTCATTATCGTGCTCATCCCTGGCGACTCTAGTATTGTCACACGGTCCCGGCTGCTGGACCGCCTCGTCAGACTGGTCGGC
GACCCAGAGATTAACGGGCCAAAGCTCACAGGAATACTGATTTCCATCCTGTCTCTGTTCGTCGAGTCCCCGGACAGCTCATCCAGCGC
ATTATTGACGACCCCGACGTGTCTATTAAGCTCGTCGAAGTCATCCCTAGTATCAACTCCGGATGCGGACTCACCTTCGCTAGTAGGGGC
GCTAGTCTCGACTCTGAGGCTGACGAGTTCTTCAAGATCGTGGACGAGGGATCTAAGGCCCAGGGCCAGCTCGGGTGGCTGGAGAACAAG
GACATTGTGGACATTGAGGTGGACGACGCCGAGCAGTTCAACATTCTCCTCGCTTCTATTCTGGCCCAGATTTGGATACTGCTCGCTAAG
GCCGTCACCGCCCCTGACACCGCCGCTGACAGTGAGATGCGCAGGTGGATTAAGTACACACAGCAGAGAAGGGTGGTCGGGGAGTTCCGC
ATGAACAAGATTTGGCTGGACATCGTGCGCAACCGGATTGCCGAGGACCTGTCTCTCAGGCGGTTCATGGTCGCCCTGATTCTGGACATC
AAGCGGTCCCCCGGAAACAAGCCTAGAATCGCCGAGATGATTTGCGACATTGACAACTACATTGTGGAGGCCGGACTGGCTTCTTTCATC
CTCACAATTAAGTTCGGAATTGAGACCATGTACCCTGCCCTGGGACTGCACGAGTTCTCCGGGGAATTGACCACAATTGAGTCTCTCATG
ATGCTGTACCAGCAGATGGGGGAGACCGCTCCATACATGGTCATCCTCGAAAACTCCGTGCAGAACAAGTTCAGTGCCGGATCTTACCCA
CTGCTGTGGTCCTACGCTATGGGCGTCGGCGTCGAGTTGGAGAACAGTATGGGCGGCCTCAACTTCGGGCGTAGCTACTTCGACCCCGCT
TACTTCCGCCTCGGGCAAGAGATGGTGCGCAGAAGTGCCGGAAAGGTCAGTAGCGCCCTCGCTGCCGAGCTGGGCATTACCAAAGAAGAG
GCCCAGCTCGTCAGTGAGATTGCTTCCAAGACCACAGAGGACCGCACCATTAGAACCGCCGGGCCAAAGCAGAGTCAGATCACCTTCCTG
CACTCTGAGCGGTCCGAGGTGACAAACCAGCAGCCACCTACAATTAACAAGCGGTCTGAGAACCCAGGCGGCGACAAGTACTCCATCCAC
TTCAACGACGAGCGGTTCAGTGGGTACACACCTGACGTGAACAGTAGTGAGTGGTCCGAGTCTAGGTACGACACCCAGACCATTCAGGAC
GACGGCAACGACGACGACAGGAAGTCTATGGAGGCTATCGCTAAGATGCGTATGCTGACAAAGATGCTGTCCCAGCCACGCACATCTGAA
GAAAGTAGTCCCGTGTACAACGACCGCGAGCTGCTGAACTAG
```

2. DNA Alignment

```
Optimized      1    ATGGCCAGTCTCCTGAAGAGCCTCACACTGTTCAAGCGCACACGCGACCAGCCACCACTG
Original       1    ATGGCTAGCCTTCTCAAGAGCCTCACACTGTTCAAGAGGACTCGGGACCAACCCCCACTT

Optimized     61    GCTTCCGGAAGTGGCGGGGCTATTAGGGGAATCAAGCACGTCATTATCGTGCTCATCCCT
Original      61    GCCTCGGGCTCCGGAGGAGCAATAAGAGGGATAAAGCATGTCATTATAGTCCTAATCCCG

Optimized    121    GGCGACTCTAGTATTGTCACACGGTCCCGGCTGCTGGACCGCCTCGTCAGACTGGTCGGC
Original     121    GGTGATTCAAGCATTGTTACAAGGTCTCGACTATTGGACAGACTTGTTAGATTGGTCGGT

Optimized    181    GACCCAGAGATTAACGGGCCAAAGCTCACAGGAATACTGATTTCCATCCTGTCTCTGTTC
Original     181    GATCCGGAAATCAACGGACCTAAATTAACCGGGATTTTAATCAGTATCCTCTCCTTGTTC

Optimized    241    GTCGAGTCCCCCGGACAGCTCATCCAGCGCATTATTGACGACCCCGACGTGTCTATTAAG
Original     241    GTGGAATCCCCTGGACAGTTGATCCAAAGGATCATAGACGACCCTGATGTGAGCATCAAG

Optimized    301    CTCGTCGAAGTCATCCCTAGTATCAACTCCGGATGCGGACTCACCTTCGCTAGTAGGGGC
Original     301    TTAGTAGAGGTAATCCCAAGCATCAACTCTGGTTGTGGTCTTACATTTGCATCCAGAGGA

Optimized    361    GCTAGTCTCGACTCTGAGGCTGACGAGTTCTTCAAGATCGTGGACGAGGGATCTAAGGCC
Original     361    GCAAGTTTGGATTCTGAGGCAGATGAGTTCTTCAAAATTGTAGACGAAGGGTCGAAAGCT

Optimized    421    CAGGGCCAGCTCGGGTGGCTGGAGAACAAGGACATTGTGGACATTGAGGTGGACGACGCC
Original     421    CAAGGACAATTAGGCTGGTTGGAGAATAAGGATATTGTAGACATAGAAGTTGATGATGCT

Optimized    481    GAGCAGTTCAACATTCTCCTCGCTTCTATTCTGGCCCAGATTTGGATACTGCTCGCTAAG
```

```
Original      481   GAGCAATTCAATATATTGCTAGCTTCCATCTTGGCCCAAATTTGGATCCTGCTCGCTAAA

Optimized     541   GCCGTCACCGCCCCTGACACCGCCGCTGACAGTGAGATGCGCAGGTGGATTAAGTACACA
Original      541   GCAGTGACTGCTCCTGATACTGCAGCCGACTCGGAAATGAGGAGATGGATTAAGTATACC

Optimized     601   CAGCAGAGAAGGGTGGTCGGGGAGTTCCGCATGAACAAGATTTGGCTGGACATCGTGCGC
Original      601   CAACAGAGACGTGTGGTCGGGGAATTTAGAATGAACAAAATCTGGCTTGATATTGTTAGA

Optimized     661   AACCGGATTGCCGAGGACCTGTCTCTCAGGCGGTTCATGGTCGCCCTGATTCTGGACATC
Original      661   AACAGGATTGCTGAGGACTTATCTTTGAGGCGATTCATGGTGGCACTCATCTTGGACATC

Optimized     721   AAGCGGTCCCCCGGAAACAAGCCTAGAATCGCCGAGATGATTTGCGACATTGACAACTAC
Original      721   AAACGATCCCCAGGGAACAAGCCTAGAATTGCTGAAATGATTTGTGATATAGATAACTAC

Optimized     781   ATTGTGGAGGCCGGACTGGCTTCTTTCATCCTCACAATTAAGTTCGGAATTGAGACCATG
Original      781   ATTGTTGAAGCTGGATTAGCTAGTTTCATCTTAACTATCAAATTTGGCATTGAAACTATG

Optimized     841   TACCCTGCCCTGGGACTGCACGAGTTCTCCGGGGAATTGACCACAATTGAGTCTCTCATG
Original      841   TATCCGGCTCTCGGGTTGCATGAGTTTTCCGGAGAGTTAACAACTATTGAATCCCTTATG

Optimized     901   ATGCTGTACCAGCAGATGGGGGAGACCGCTCCATACATGGTCATCCTCGAAAACTCCGTG
Original      901   ATGCTATATCAACAGATGGGTGAAACAGCACCGTACATGGTTATTCTGGAAAATTCTGTT

Optimized     961   CAGAACAAGTTCAGTGCCGGATCTTACCCACTGCTGTGGTCCTACGCTATGGGCGTCGGC
Original      961   CAGAACAAATTTAGTGCAGGATCCTACCCACTGCTCTGGAGTTATGCTATGGGAGTTGGT

Optimized    1021   GTCGAGTTGGAGAACAGTATGGGCGGCCTCAACTTCGGGCGTAGCTACTTCGACCCCGCT
Original     1021   GTTGAACTTGAAAACTCCATGGGAGGGTTAAATTTCGGTAGATCCTACTTTGATCCGGCC

Optimized    1081   TACTTCCGCCTCGGGCAAGAGATGGTGCGCAGAAGTGCCGGAAAGGTCAGTAGCGCCCTC
Original     1081   TATTTCAGGCTCGGGCAAGAAATGGTGAGAAGATCTGCCGGTAAAGTAAGCTCTGCACTT

Optimized    1141   GCTGCCGAGCTGGGCATTACCAAAGAAGAGGCCCAGCTCGTCAGTGAGATTGCTTCCAAG
Original     1141   GCCGCCGAGCTTGGCATCACCAAGGAAGAGGCTCAGCTAGTGTCAGAAATAGCATCCAAG

Optimized    1201   ACCACAGAGGACCGCACCATTAGAACCGCCGGGCCAAAGCAGAGTCAGATCACCTTCCTG
Original     1201   ACAACGGAGGACCGGACGATTCGCACTGCTGGTCCCAAGCAATCTCAAATCACTTTTCTG

Optimized    1261   CACTCTGAGCGGTCCGAGGTGACAAACCAGCAGCCACCTACAATTAACAAGCGGTCTGAG
Original     1261   CACTCAGAAAGATCCGAAGTCACTAATCAACAACCCCCAACTATCAACAAGAGGTCCGAA

Optimized    1321   AACCCAGGCGGCGACAAGTACTCCATCCACTTCAACGACGAGCGGTTCAGTGGGTACACA
Original     1321   AACCCAGGAGGAGACAAATACTCCATCCACTTCAATGATGAACGATTTTCAGGGTACACC

Optimized    1381   CCTGACGTGAACAGTAGTGAGTGGTCCGAGTCTAGGTACGACACCCAGACCATTCAGGAC
Original     1381   CCTGATGTCAATAGCTCCGAATGGAGTGAATCACGCTATGATACCCAGACCATTCAAGAT

Optimized    1441   GACGGCAACGACGACGACAGGAAGTCTATGGAGGCTATCGCTAAGATGCGTATGCTGACA
Original     1441   GATGGAAACGACGATGACCGGAAATCGATGGAAGCAATCGCCAAGATGAGAATGCTTACT
```

```
Optimized     1501    AAGATGCTGTCCCAGCCACGCACATCTGAAGAAAGTAGTCCCGTGTACAACGACCGCGAG
Original      1501    AAGATGCTCAGTCAACCTAGAACCAGTGAAGAGAGTTCTCCTGTCTATAATGATAGAGAG

Optimized     1561    CTGCTGAACTAG
Original      1561    CTACTCAATTAA
```

3. Supplementary - Codon frequency table

Species: Neovison vison

TTT	0.405	TTC	0.595	TTA	0.054	TTG	0.123
TCT	0.179	TCC	0.246	TCA	0.129	TCG	0.050
TAT	0.392	TAC	0.608	TAA	0.263	TAG	0.368
TGT	0.444	TGC	0.556	TGA	0.368	TGG	1.000
CTT	0.127	CTC	0.238	CTA	0.056	CTG	0.402
CCT	0.292	CCC	0.373	CCA	0.248	CCG	0.087
CAT	0.395	CAC	0.605	CAA	0.237	CAG	0.763
CGT	0.093	CGC	0.168	CGA	0.101	CGG	0.229
ATT	0.326	ATC	0.553	ATA	0.121	ATG	1.000
ACT	0.184	ACC	0.414	ACA	0.264	ACG	0.137
AAT	0.430	AAC	0.570	AAA	0.378	AAG	0.622
AGT	0.151	AGC	0.244	AGA	0.217	AGG	0.192
GTT	0.145	GTC	0.253	GTA	0.101	GTG	0.501
GCT	0.267	GCC	0.447	GCA	0.181	GCG	0.105
GAT	0.385	GAC	0.615	GAA	0.408	GAG	0.592
GGT	0.141	GGC	0.376	GGA	0.252	GGG	0.231

4. Supplementary - Protein Alignment

```
Optimized      1    MASLLKSLTLFKRTRDQPPLASGSGGAIRGIKHVIIVLIPGDSSIVTRSRLLDRLVRLVG
Original       1    MASLLKSLTLFKRTRDQPPLASGSGGAIRGIKHVIIVLIPGDSSIVTRSRLLDRLVRLVG

Optimized     61    DPEINGPKLTGILISILSLFVESPGQLIQRIIDDPDVSIKLVEVIPSINSGCGLTFASRG
Original      61    DPEINGPKLTGILISILSLFVESPGQLIQRIIDDPDVSIKLVEVIPSINSGCGLTFASRG

Optimized    121    ASLDSEADEFFKIVDEGSKAQGQLGWLENKDIVDIEVDDAEQFNILLASILAQIWILLAK
Original     121    ASLDSEADEFFKIVDEGSKAQGQLGWLENKDIVDIEVDDAEQFNILLASILAQIWILLAK

Optimized    181    AVTAPDTAADSEMRRWIKYTQQRRVVGEFRMNKIWLDIVRNRIAEDLSLRRFMVALILDI
Original     181    AVTAPDTAADSEMRRWIKYTQQRRVVGEFRMNKIWLDIVRNRIAEDLSLRRFMVALILDI

Optimized    241    KRSPGNKPRIAEMICDIDNYIVEAGLASFILTIKFGIETMYPALGLHEFSGELTTIESLM
Original     241    KRSPGNKPRIAEMICDIDNYIVEAGLASFILTIKFGIETMYPALGLHEFSGELTTIESLM

Optimized    301    MLYQQMGETAPYMVILENSVQNKFSAGSYPLLWSYAMGVGVELENSMGGLNFGRSYFDPA
Original     301    MLYQQMGETAPYMVILENSVQNKFSAGSYPLLWSYAMGVGVELENSMGGLNFGRSYFDPA

Optimized    361    YFRLGQEMVRRSAGKVSSALAAELGITKEEAQLVSEIASKTTEDRTIRTAGPKQSQITFL
Original     361    YFRLGQEMVRRSAGKVSSALAAELGITKEEAQLVSEIASKTTEDRTIRTAGPKQSQITFL

Optimized    421    HSERSEVTNQQPPTINKRSENPGGDKYSIHFNDERFSGYTPDVNSSEWSESRYDTQTIQD
Original     421    HSERSEVTNQQPPTINKRSENPGGDKYSIHFNDERFSGYTPDVNSSEWSESRYDTQTIQD

Optimized    481    DGNDDDRKSMEAIAKMRMLTKMLSQPRTSEESSPVYNDRELLN!
Original     481    DGNDDDRKSMEAIAKMRMLTKMLSQPRTSEESSPVYNDRELLN!
```
